# Finite Element-Based Personalized Simulation of Duodenal Hydrogel Spacer: Spacer Location Dependent Duodenal Sparing and a Decision Support System for Spacer-Enabled Pancreatic Cancer Radiation Therapy

**DOI:** 10.3389/fonc.2022.833231

**Published:** 2022-03-24

**Authors:** Hamed Hooshangnejad, Sina Youssefian, Amol Narang, Eun Ji Shin, Avani Dholakia Rao, Sarah Han-Oh, Todd McNutt, Junghoon Lee, Chen Hu, John Wong, Kai Ding

**Affiliations:** ^1^Department of Biomedical Engineering, Johns Hopkins School of Medicine, Baltimore, MD, United States; ^2^Department of Radiation Oncology and Molecular Radiation Sciences, Johns Hopkins School of Medicine, Baltimore, MD, United States; ^3^Department of Gastroenterology, Johns Hopkins School of Medicine, Baltimore, MD, United States; ^4^Division of Biostatistics and Bioinformatics, Sidney Kimmel Comprehensive Cancer Center, Johns Hopkins School of Medicine, Baltimore, MD, United States

**Keywords:** FEMOSSA, Bayesian-based decision support system, finite element-based simulation, spacer-enabled pancreatic radiotherapy, personalized duodenal hydrogel spacer

## Abstract

**Purpose:**

Pancreatic cancer is the fourth leading cause of cancer-related death, with a very low 5-year overall survival rate (OS). Radiation therapy (RT) together with dose escalation significantly increases the OS at 2 and 3 years. However, dose escalation is very limited due to the proximity of the duodenum. Hydrogel spacers are an effective way to reduce duodenal toxicity, but the complexity of the anatomy and the procedure makes the success and effectiveness of the spacer procedure highly uncertain. To provide a preoperative simulation of hydrogel spacers, we presented a patient-specific spacer simulator algorithm and used it to create a decision support system (DSS) to provide a preoperative optimal spacer location to maximize the spacer benefits.

**Materials and Methods:**

Our study was divided into three phases. In the validation phase, we evaluated the patient-specific spacer simulator algorithm (FEMOSSA) for the duodenal spacer using the dice similarity coefficient (DSC), overlap volume histogram (OVH), and radial nearest neighbor distance (RNND). For the simulation phase, we simulated four virtual spacer scenarios based on the location of the spacer in para-duodenal space. Next, stereotactic body radiation therapy (SBRT) plans were designed and dosimetrically analyzed. Finally, in the prediction phase, using the result of the simulation phase, we created a Bayesian DSS to predict the optimal spacer location and biological effective dose (BED).

**Results:**

A realistic simulation of the spacer was achieved, reflected in a statistically significant increase in average target and duodenal DSC for the simulated spacer. Moreover, the small difference in average mean and 5th-percentile RNNDs (0.5 and 2.1 mm) and OVH thresholds (average of less than 0.75 mm) showed that the simulation attained similar separation as the real spacer. We found a spacer-location-independent decrease in duodenal V20Gy, a highly spacer-location-dependent change in V33Gy, and a strong correlation between L1cc and V33Gy. Finally, the Bayesian DSS predicted the change in BED with a root mean squared error of 3.6 Gys.

**Conclusions:**

A duodenal spacer simulator platform was developed and used to systematically study the dosimetric effect of spacer location. Further, L1cc is an informative anatomical feedback to guide the DSS to indicate the spacer efficacy, optimum location, and expected improvement.

## 1 Introduction

Pancreatic cancer is the fourth leading cause of cancer-related death and the 12th most common malignancy in the US, with nearly 60,000 cases each year and only less than 10% 5-year overall survival rate (1). More than one-third of the patients present with local and local/regional metastasis stage and are at great risk of distant progression (1–3). Therefore, local control (LC) is of great importance for these patients. Radiation therapy (RT), as a local-regional anticancer treatment, is an effective way to achieve LC, and using the dose escalation with intensity-modulated radiotherapy (IMRT) and stereotactic body radiation therapy (SBRT) can improve the RT outcome (4–7).

Recent studies have shown that RT together with dose escalation increases the OS at 2 years from 19% to 36%, and at 3 years from 9% to 31% (8, 9). Reaching the biologically effective dose (BED_10_) of 70 Gy can considerably improve LC and overall survival rate (4, 10–15). However, the major concern with dose escalation is the toxicity of adjacent organs at risk (OARs), namely, stomach, bowel, and primarily duodenum, due to its proximity to the pancreas. MRI-guided RT is now used in some treatment units, in which high tumor doses can be delivered while still meeting the toxicity constraints of OAR (16, 17). However, a lack of compatibility with the current treatment system and time-consuming workflow limits its application. Achieving safe dose escalation for daily treatment using MRI information needs a new daily MRI image, which in turn triggers a new and longer and undesirable treatment plan (30 to 40 min longer). Unfortunately, even the detection of intra-fraction anatomic variation during the lengthy treatment would rarely lead to further intervention due to time constraints.

Another possible solution to deliver a high radiation dose while sparing the radiosensitive organs is the insertion of a spacer to increase the separation between the tumor and OARs. Duodenal hydrogel spacer implantation is shown to be an effective way to increase the separation between the tumor and duodenum to decrease the duodenal dose and toxicity (13, 18–26). The previous studies demonstrated that the injection of a rectal hydrogel spacer comes with many risks such as infection, inflammation, soft-tissue wall infiltration, and uncertainty in outcome of the procedure (27–29). Moreover, the misplaced hydrogel may decrease the efficacy and cause further discomfort for the patient (29). Similar risks are associated with the duodenal spacer for pancreatic cancer patients which makes the effectiveness of the spacer insertion procedure uncertain.

**Figure 1:**
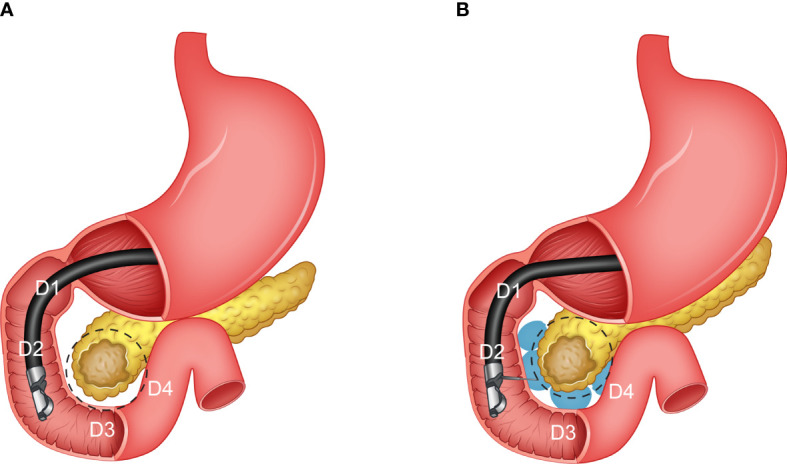
An illustration of the pancreas and different sections of the duodenum (C loop). **(A)** Before the hydrogel injection (pre-injection), and **(B)** after the hydrogel injection (post-injection).

Additionally, the hard-to-reach location of the duodenum pancreas interface considerably increases the complexity of the procedure and chance of failure. Moreover, the duodenal hydrogel spacer system (TraceIT Tissue Marker; Boston Scientific, Marlborough, MA), at the current stage, is only designed to be used for separation of the head of the pancreas (HOP) and duodenum (**Figure 1**). However, stomach and bowel can also be major dose-limiting OARs for pancreatic RT. Thus, in such a case, the spacer may not have enough, or even, any benefits at all. Thus, the lack of a predictive model of spacer placement and dosimetric benefits may limit the optimal use of a duodenal spacer in practice.

The purpose of this study is to provide a preoperative decision support system by performing a systematic study of the different scenarios of spacer location in the duodenal loop. To do so, we took advantage of our in-house, anatomical-based and patient-specific hydrogel spacer simulation algorithm, FEMOSSA, to simulate the different scenarios of hydrogel spacer placement (30, 31). Next, we designed SBRT plans and used the result of dosimetric analysis of the RT plans to create a predictive decision support system (DSS) for the duodenal hydrogel injection procedure.

The DSS aims at helping the physician better decide whether the spacer procedure is beneficial, and FEMOSSA provides a preoperative simulation of spacer placement that can be used for a detailed examination of dose distribution and dosimetric analysis. If the spacer location is approved, the preoperative simulation guides the spacer insertion procedure. Hence, we hypothesize that the efficacy of the duodenal spacer highly depends on the duodenum-target geometry, and the DSS and FEMOSSA can personalize and optimize the hydrogel injection procedure and thus maximize the benefits and minimize the risks and uncertainties. As a result, we believe that this study is a realization of precision medicine in pancreatic cancer treatment (32).

## 2 Materials and Methods

We divided our work into three phases: validation phase, simulation phase, and prediction phase. Due to the very limited number of clinical cases of the duodenal spacer, to design and create a reliable predictive decision support system, we first extended the application of our hydrogel spacer simulation algorithm, FEMOSSA, to simulate different scenarios of spacer placement. Thus, as the first step, in the validation phase, we fine-tuned the FEMOSSA parameters to spacer insertion in the duodenum–pancreas interface problem and validated the simulation performance with the pair pre–post-injection data. FEMOSSA has already been validated for rectal spacer simulation and has shown strong performance in providing a patient-specific simulation of rectal hydrogel insertion compared to other studies (30, 33). Previously, our group also simulated the virtual spacer by shifting the structures (34), and more recently, another group used contour overriding (19). To the best of our knowledge, FEMOSSA is the first of its kind to provide an anatomical-based and patient-specific simulation of duodenal hydrogel spacers.

In the simulation phase, FEMOSSA was used to simulate four different spacer insertion scenarios to study the correlation between spacer location and benefits. Finally, in the prediction phase, SBRT plans were created for all scenarios and analyzed from the dosimetric point of view to create the DSS. For the validation phase, the spacer distribution was determined from a rigid registration of pre-and postinjection; however, for the simulation phase, by taking advantage of the FEMOSSA built-in user interface, we simulated the virtual spacer in various sections of the duodenal C-loop. **Table 1** shows an overview of the three phases of the study.

**Table 1:** An overview of the three phases of the study.

	Validation phase	Simulation phase	Prediction phase
**Purpose**	Validating the virtual spacer platform	Studying the correlation of spacer location and benefits	Designing a decision support system for duodenal spacer
**Data**	4 cases with pre–post-injection pair scans	20 cases of pancreatic cancer patients	Dosimetric analysis from simulation phase
**Method**	Post-injection spacer distribution simulated in pre-injection	4 scenarios of virtual spacer were simulated, and RT plans designed	A Bayesian-based predictive model was created
**Primary result**	FEMOSSA was fine-tuned for virtual duodenal spacer	Spacer benefits highly depend on spacer location	The model predicts the optimal scenario and expected benefits of spacer

### 2.1 Data Collection and Preparation

Data from two cadavers and 20 patients, a total of 22 cases, were used for this study. Two cadavers and two patients with pre–postinjection scans available were used for the validation phase, and the pre-injection scans from the 20 patients (including the pre-injection scans from two patients used for validation) were included in the simulation and prediction phases of the study. Organ contours were delineated by clinicians using Varian Velocity (Varian Medical Systems, Palo Alto, CA). All scans were acquired with 2-mm slice thickness, 120 kVp, 200 mA, and 50 cm field of view.

### 2.2 Validation Phase

FEMOSSA parameters were, first, fine-tuned, and then the simulation result was validated on data from two cadavers and two patients that have been injected with duodenal hydrogel. Since, in this study, we used the same principles and only fine-tuned FEMOSSA for the duodenal spacer, for more detail on the different components of FEMOSSA we encourage the readers to refer to our previous study (Hamed 30). Similar figures of merit as our previous study were used to evaluate the duodenal spacer from different aspects: (1) dice similarity coefficient (DSC) between the target and duodenum in postinjection (ground truth) and post-simulation scans, (2) radial definition of nearest neighbor distance (RNND), adapted to the C-loop-like anatomy of the duodenal loop, and (3) overlapped volume histogram (OVH) *L*_1_*_CC_*, *L*_3_*_CC_*, *L*_5_*_CC_*, *L*_10_*_CC_*, and *L*_20_*_CC_* defined as the amount of uniform expansion of the target to have 1-, 3-, 5-, 10-, and 20-cc-volume overlaps with the duodenum (30).

#### 2.2.1 Finite Element Model Generation

Here, we summarized the steps to create the FE model from the original contours. The generation of the FE model began by converting the 3D binary masks to triangular surface mesh. The surface mesh was smoothed using the volume-preserving Laplacian smoothing algorithm. A 3D four-node tetrahedral was used to create elements bounded to the triangular surface mesh and thus a volume mesh of the structures. To have an accurate representation of hollow organs, like the duodenum, we developed an algorithm that creates a volume 3D mesh bounded to two triangular surface meshes 2 to 3 mm away from each other. The thickness of 2 to 3 mm for the duodenum is chosen based on measurements from previous clinical studies (35–37). We turned this complex physical phenomenon into a more manageable and practically solvable problem by using an innovative, simplified, and yet realistic definition. We defined the spacer placement procedure as a translation of hydrogel distribution assembly from an initial position, tangent to the surface of ROIs, toward the final, desired spacer location that on its way pushes the proximal ROI surface and deforms them.

We ensure the well-posed definition of the FE problem by using boundary conditions inspired by the anatomy of the duodenum–pancreas interface. Comparing the pre-injection and postinjection scans revealed that the inferior surface of the horizontal part of the duodenum (D3) relatively stays in the same position. On the other hand, the descending and ascending parts of the duodenum (D2 and D4) move considerably. Due to the higher stiffness of the stomach and sphincter, the movement of the duodenum section immediately after the stomach (D1) is limited. Accordingly, the mesh nodes corresponding to the inferior wall of the D3 and the nodes on the duodenum mesh within 2-mm distance from the stomach were bound to mimic these restrictions. In the case of the target structure (HOP), no global movement, but rather a local deformation of the HOP–duodenum interface, was observed. Thus, we fixated the superior and inferior margins of HOP mesh, preventing global movements while allowing local deformation of the structure.

#### 2.2.2 Finite Element Analysis

For the validation cases, the postinjection scans were used as the ground truth. To determine the spacer distribution in the pre-injection scan, we rigidly registered the postinjection scan to pre-injection. The distribution of the spacer in the rigidly registered scan was used for virtual spacer simulation in the pre-injection set. The FE model was, then, analyzed and solved for nodes’ translation using the ABAQUS software package. The analysis was done on a Dell XPS 15, 7590, equipped with 2.4 GHz Intel Core i9, and 32 Gigabytes RAM. Finally, the results of FE analysis were interpreted as a deformation vector field that was applied to the pre-injection scan and structure set to create the post-simulation scan and structures.

#### 2.2.3 Model Evaluation

Three figures of merit were used to evaluate the duodenal spacer from different aspects. First is the dice similarity coefficient (DSC) for target and duodenum postinjection (ground truth) and post-simulation masks. The DSC provides an insight into the general similarity of the 3D structures. However, the main goal of FEMOSSA is to simulate the separation of ROIs rather than producing the same exact contours, which is the purpose of the registration task. Thus, to evaluate the separation from a 3D point of view, we compared the OAR and target-overlapped volume histogram (OVH) between the post-simulation and postinjection. We chose five points on the OVH curve, *L*_1_*_CC_*, *L*_3_*_CC_*, *L*_5_*_CC_*, *L*_10_*_CC_*, and *L*_20_*_CC_* -the uniform expansion of the target that overlaps with 1-, 3-, 5-, 10-, and 20-cc volume of OAR, respectively.

While OVH provides a volumetric 3D evaluation of the increase in separation, the radial nearest neighbor distance (RNND) gives a 2D evaluation of the separation. For any two given structures, RNND measures the closest distance from every point on one structure’s margin to all the points on the margin of the other structure that fall in the same 3D spatial angle range (angle bin). Since the stomach and adjacent duodenum (D1) remain relatively in the same location compared to the surrounding structures, it was used as the origin for angle calculation. For every angle bin, a distribution of RNNDs was obtained, and the mean and 5th-percentile values were used as the representative values.

### 2.3 Simulation Phase

#### 2.3.1 Virtual Spacer Simulation Scenarios

The pre-injection scan from 20 cases (Scenario Zero, S0) was augmented with three virtual spacer scenarios based on the involvement of the duodenum–target interface: between the target and D1–D2 (Scenario one, S1), D1–D2 and D3 (Scenario two, S2), and lastly, D1–D2, D3, and D4 (Scenario three, S3). Based on our experience in early trials, the injected hydrogel volume for each section was limited to less than 10 ml (20). SBRT plans were designed for the four scenarios and then analyzed to study the correlation between spacer location and benefits.

#### 2.3.2 SBRT Planning

A total of 80 (20 cases and each case four scenarios) volumetric modulated arc therapy SBRT plans (33 Gy in 5 fractions) were designed according to the RT planning protocol in our institute. The gross target volume (GTV) was expanded by 3 mm to get mock GTV (GTV-multabc) from multiple CTs under active breath control. The GTV-multabc was further expanded by 2 mm to get the planning target volume (PTV). For further details, please refer to our previous study (14, 38).

The SBRT planning objectives and constraints were as follows: at least 90% of PTV volume receive 33 Gy, 100% of PTV volume receive 25 Gy, less than 1 cc of PTV volume receive ≥42.9 Gy, at least 95% of GTV-multabc volume receive 33 Gy, 100% of GTV volume receive 33, less than 25% of kidney volume receive ≥12 Gy, less than 50% of liver volume receive ≥12 Gy, less than 20 cc of duodenum, stomach, and bowel volume receive ≥20 Gy, less than 1 cc of duodenum, stomach, and bowel volume receive ≥33 Gy, and less than 1 cc of spinal cord volume receive ≥8 Gy. To avoid any planning bias, the planning parameters, namely, the number of beams, number of iterations, and objective functions, were identical for all the plans. Plans were optimized on the Pinnacle treatment planning system (Philips Radiation Oncology Systems, Milpitas, CA).

### 2.4 Prediction Phase

#### 2.4.1 Predictive Decision Support System Design

A DSS was designed to determine (a) which OARs are the dose-limiting structures, (b) whether the patient will benefit from spacer insertion procedure, (c) how much separation is needed to achieve the desired BED, and (d) depending on patients’ anatomy and dose-limiting OARs, predicted increase in *maxBED*, and thus the effectiveness of the spacer placement procedure. *maxBED* was defined as the BED value corresponding to the maximum achievable dose escalation by scaling the plan while no OAR constraints were violated. BED was calculated with α/β ratio of 10 for the tumor.

#### 2.4.2 Decision Support System Implementation

**Figure 2:**
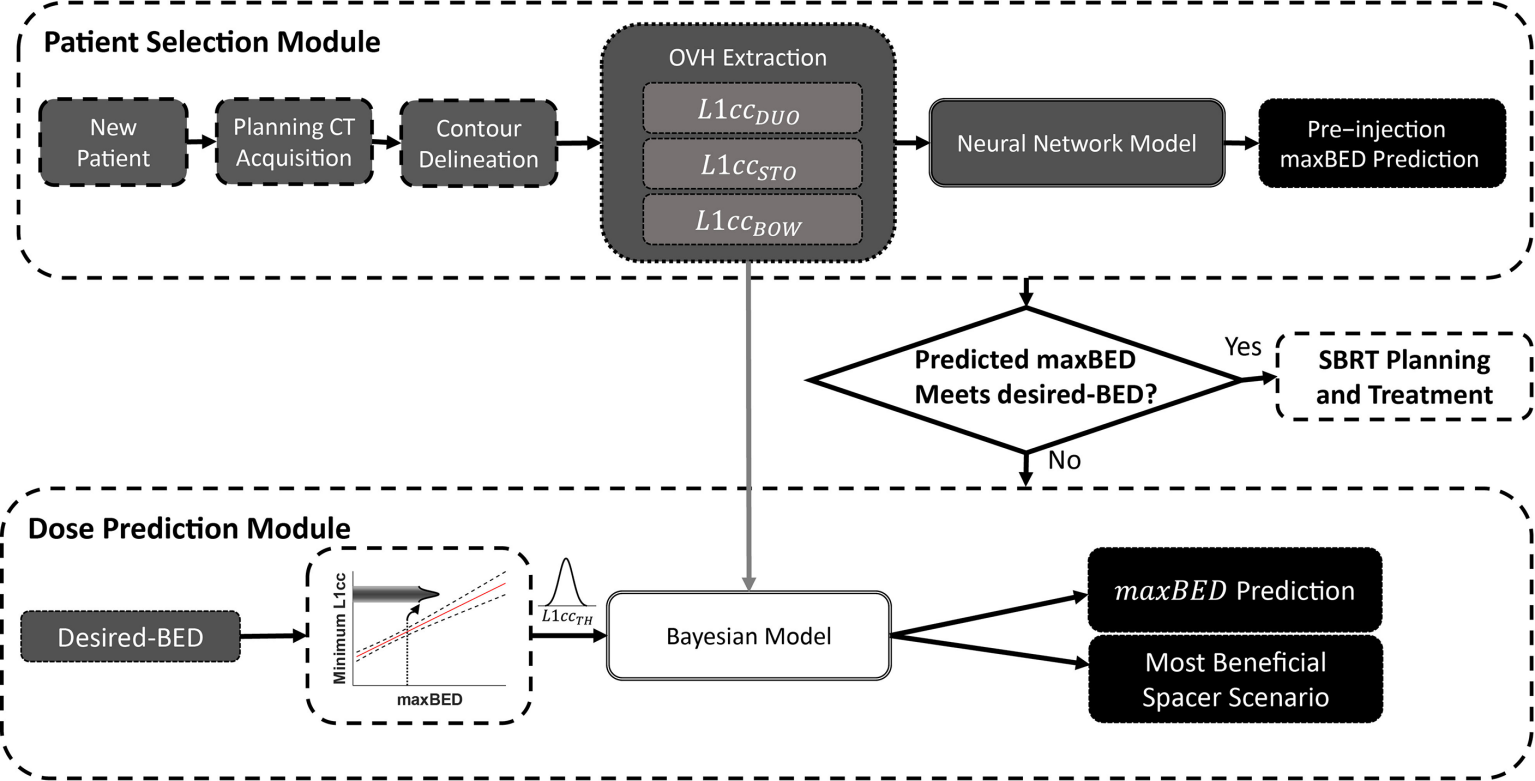
The overview of the decision support system to predict the optimal location of the spacer and maximum achievable BED.

As shown in **Figure 2**, the DSS is composed of three main components: (1) a neural network (NN) to predict the pre-injection *maxBED* using pre-injection *L*1*_CC_* anatomical information; (2) a linear regression model between desired-BED and minimum required *L*1*_CC_*, and (3) a Bayesian regression model to predict the postinjection benefits of the spacer. For every new patient after the organs are delineated on the initial scan, OVH distances are extracted. The NN predicts the pre-injection *maxBED* using the *L*1*_CC_* for the three proximal OARs (duodenum, stomach, and bowel).

To decide whether the patient benefits from the spacer insertion, a desired BED value is needed as a reference value for making the decision. The corresponding *L*1*_CC_* $\left(L1_{CC_{TH}}\right)$ to the desired BED is used to determine which OAR(s) and section of the duodenal loop are dose-limiting. In addition to the duodenum, stomach and bowel can potentially severely hinder achieving plan objectives due to their proximity to the target; however, unlike in the duodenum, spacer insertion in the pancreaticoduodenal space does not reduce the receiving dose to these ROIs.

For any given $L1_{CC_{TH}}$ , there are three possible scenarios: (P1) only $L1_{CC_{duodenum}}$ is less than $L1_{CC_{TH}}$ , (P2) $L1_{CC_{duodenum}}$ and one or both of $L1_{CC_{stomach}}$ and $L1_{CC_{bowel}}$ are less than $L1_{CC_{TH}}$ , and (P3) none or only $L1_{CC_{stomach}}$ and/or $L1_{CC_{bowel}}$ are less than $L1_{CC_{TH}}$ . The change in *maxBED* with spacer insertion (Δ*maxBED*) highly depends on the geometry of proximal OARs (P1–P3). P1 is the most beneficial case for spacer insertion, as the spacer insertion directly affects duodenal *L*1*_CC_* $\left(L1_{CC_{DUO}}\right)$ . On the other hand, there is less improvement for P2 as the duodenum is not the only limiting OAR. For the P3 scenario, however, spacer insertion is not beneficial, because *maxBED* is limited by the stomach and/or bowel, but the spacer can only spare the duodenum.

We created a Bayesian multiple linear regression model using MATLAB built-in function *bayeslm*. The input to the model is the spacer-induced change in duodenum separation $\left(\Delta L1_{CC_{DUO}}\right)$ . The output of the model is the spacer-induced change in *maxBED* for two possibilities P1 and P2 (Δ*maxBED_Px_*, *x* = {1, 2}). Δ*maxBED_P_*_1_ was defined as subtraction of pre-injection *maxBED* from post-simulation maximum achievable BED while only duodenum constraints are met. Similarly, Δ*maxBED_P_*_2_ was defined as subtraction of pre-injection *maxBED* from post-simulation maximum achievable BED while all constraints for the three proximal OARs are met.

We defined the Bayesian linear regression model as


$$\Delta maxBED_{Px}=\beta_{0}+\beta_{1}X+\beta_{2}\Delta L1_{CC_{DUO}}+\beta_{3}X\Delta L1_{CC_{DUO}}+\epsilon$$

where *X* is 0 for P1 and 1 for P2, and ϵ is the stochastic error term. The model creates an empirical distribution of prior probabilities for the model parameters using the Gibbs (Markov chain Monte Carlo algorithm) sampling method (10,000 draws). As a result, instead of point estimation, for each parameter an empirical posterior distribution was obtained and therefore incorporates the inherent high variability of the data.

To make a prediction using the model, DSS compares the *L*1*_CC_* of each proximal OAR with a minimum required distance $L1_{CC_{TH}}$ to find the limiting OARs. If the predicted pre-injection *maxBED* is less than desired-BED, depending on the patient-specific dose-limiting OARs, the Bayesian model predicts the change in *maxBED* after spacer placement. 10,000 samples from the posterior distribution of linear regression parameters, and the normal distribution of $L1_{CC_{TH}}$ (fitted to prediction mean and 95% confidence interval), were fed to the Bayesian regression model to generate a posterior probability of *maxBED*. For prediction, the input to the model is the amount separation needed, the subtraction of pre-injection $L1_{CC_{DUO}}$ from $L1_{CC_{TH}}$ . The final output of the model is the maximum likelihood estimation of Δ*maxBED*.

### 2.5 Statistical Analysis

Using a pairwise permutation test (n = 1,000), we tested the relationship between pre-injection, post-simulation pair, and post-simulation, postinjection DSC values. Because of the small number of subjects in the validation phase, the normality assumption was circumvented by using a non-parametric permutation test.

## 3 Results

### 3.1 Validation Results

The mean target DSC was 0.86 (range, 0.78 to 0.91) and 0.89 (range, 0.81 to 0.94) and duodenal DSC was 0.49 (range, 0.41 to 0.62) and 0.63 (range, 0.49 to 0.74) for pre–postinjection pair and post-simulation and post-injection pair, respectively. The statistically significant increase (p-value <0.01) in DSC values after simulation implies that the simulated ROIs are more similar in shape to postinjection ROIs. The low duodenal DSC is due to the highly variable shape of the duodenum.

**Figure 3:**
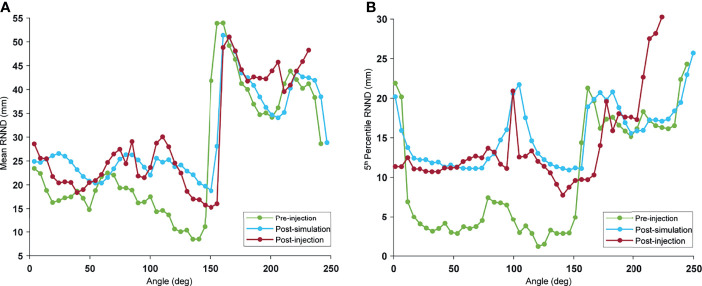
Example of RNND profile: the RNND profile was calculated for every 5° angle bin, with 0° indicating the duodenum part adjacent to stomach (D1): pre-injection (green), post-simulation (blue), and post-injection (red). Every point on the curve shows **(A)** mean, and **(B)** 5th percentile of NND values of a single-angle bin.

The mean and 5th-percentile RNND profile for the pre-injection, postinjection, and post-simulation for a typical case is illustrated in **Figure 3**. The average difference between post-simulation and postinjection mean values and 5th-percentile values over all the cases were 0.5 and 2.1 mm, respectively. As seen in **Figure 3**, there was a visible increase in RNND values because of the spacer insertion. Although the post-simulation and postinjection profiles overlapped for the most part, they diverge on the right-hand side of the curve. This is the epitome of the natural variability of the duodenum. This portion of the curve corresponds to the D4 portion of the duodenum that was not injected with hydrogel in this case. As a result, the separation did not arise from the hydrogel and, therefore, was not captured by simulation.

**Figure 4:**
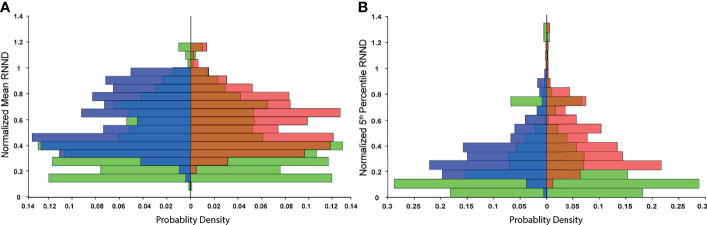
Probability distribution of RNND values over all cases: to remove the effect of biological variability, RNND values for each case are first normalized to the maximum post-injection RNND of the same case. **(A)** shows three probability distributions of normalized mean RNND values for pre-injection (green), post-simulation (blue), and post-injection (red), and **(B)** shows the probability distribution of normalized 5th percentile NNDs.

The probability distribution of RNNDs, created by pooling data and normalizing the histogram of the RNNDs over all cases, showed similar probability distribution for both postinjection and post-simulation (**Figure 4**). The absolute mean difference of OVH *L*_1_*_cc_*, *L*_3_*_cc_*, *L*_5_*_cc_*, *L*_10_*_cc_*, and *L*_20_*_cc_* between virtual and actual spacer were 0.04, 0.22,0.24, 0.34, and 0.75 mm, respectively.

### 3.2 Simulation and Planning Results

**Figure 5:**
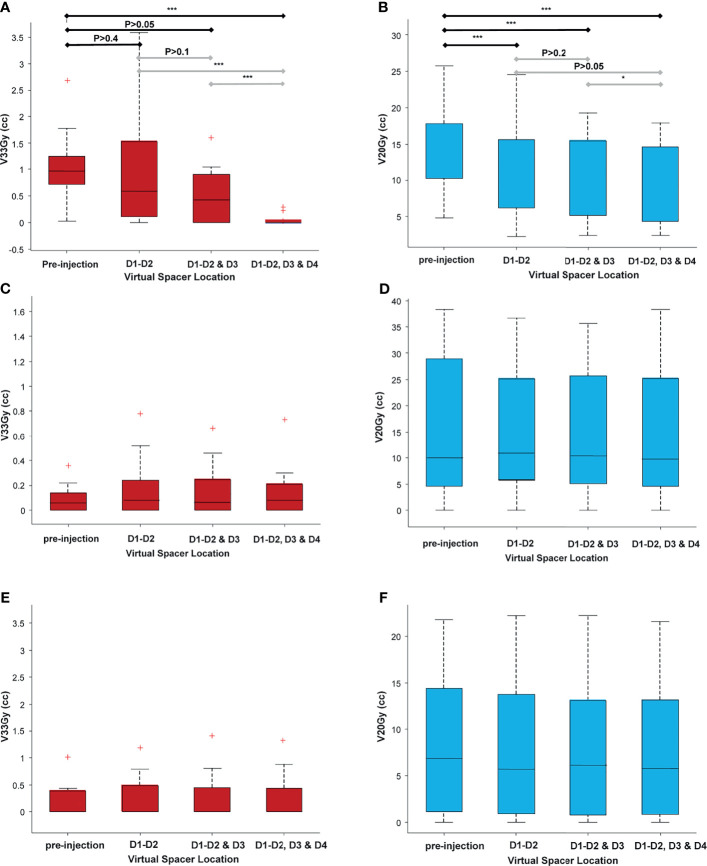
A comparison of volume receiving high and low doses for all adjacent OARs (duodenum, stomach, and bowel), broken down for different scenarios. As seen, there is a statistically significant improvement in duodenal low-dose volume [20 Gy **(B)**] independent of scenario (spacer location), as opposed to the duodenal high-dose volume [33 Gy **(A)**] that highly depends on the location of the spacer. **(C–F)** The high-dose and low-dose volumes for bowel **(C, D)** and stomach **(E, F)**. There was no statistically significant difference between the volumes among the different scenarios, indicating that the duodenal spacer placement benefits have the most effect on duodenal sparing and minimal effect on sparing the bowel and stomach. *P ≤ 0.05, **P ≤ 0.01, ***P ≤ 0.001, red + indicates outlier defined as a value that is more than 1.5 times the interquartile range away from the bottom or top of the box. The double red pluses are just two outlier close to each other.

Due to the proximity of OARs, not all plans could achieve the 95% PTV coverage (clinical goal) while meeting all OAR constraints. To make the plans comparable, they were scaled to achieve 95% <*PTV_V_*_33_*_Gy_* <96%. **Figure 5** shows the duodenal V33Gy (**Figure 5A**) and V20Gy (**Figure 5B**) values broken down by the scenarios. As seen in **Figure 5B**, there was an improvement in duodenal low-dose volume (V20Gy) independent of spacer location; however, for high-dose volume, the optimum location of the spacer highly depended on the patient’s anatomy, as no significant difference between scenarios was seen (**Figure 5A**). The S3 scenario has significantly lower V33Gy compared to all other scenarios since the full duodenal loop interface was separated from the target by the spacer. There was no significant difference between scenarios for stomach and bowel V33Gy and V20Gy, confirming the fact that duodenal spacer insertion does not increase the stomach and bowel sparing (**Figures 5C–F**).

**Figure 6:**
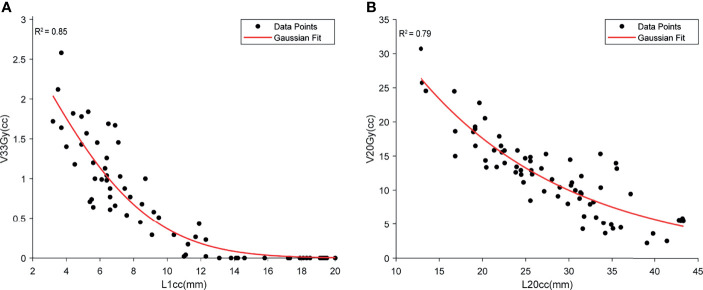
The demonstration of the relationship between high-dose duodenal volume and L1cc **(A)** and low-dose duodenal volume and L20cc **(B)**. Both Gaussian models show a high correlation between the duodenal volume and OVH distances.

A high correlation was found between duodenal L1cc and V33Gy (*r*^2^ = 0.85). A Gaussian fit was used to capture both the volumetric (power) relationship and the non-negative nature of V33 Gy. Based on the fitted model, L1cc >7 mm achieved the clinical constraint of duodenal V33Gy <1 cc, and L1cc >14 mm, resulting in V33Gy = 0 (**Figure 6A**). V20Gy and L20cc were also highly correlated (Gaussian fit *r*^2^ = 0.79), and L20cc >17 mm corresponds to V20Gy <20 cc (**Figure 6B**).

### 3.3 Decision Support System Prediction Results

**Figure 7:**
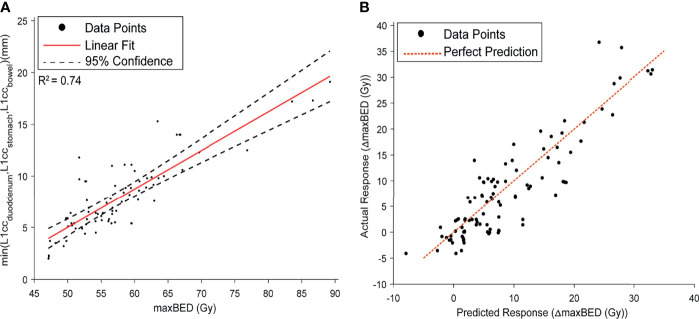
**(A)** the Linear model fit between *maxBED* and *minL*1*_CC_*. A high correlation was found between the two variables. **(B)** The prediction performance curve for the Bayesian model, predicting the Δ*maxBED*.

The NN model root mean squared prediction error for the pre-injection *maxBED* was 2.7 and 3.1 Gy for the training and test data, respectively. Moreover, we found a high linear correlation between *maxBED* and the minimum of OARs L1cc (*minL*1*_CC_*) shown in **Figure 7A** (*r*^2^ = 0.74). The Bayesian predictive model root mean squared prediction error for Δ*maxBED* was 2.7 and 3.6 Gy, for the train and test data, respectively (**Figure 7B**). Finally, our model suggests that for 70 Gy BED an L1cc of 12.4 mm (95% confidence interval, 11.5 mm, 13.3 mm) is required.

## 4 Discussion

In this study, we presented a new application for FEMOSSA as a physical-based, patient-specific spacer simulation algorithm for the duodenal hydrogel spacer. We have also used FEMOSSA for the simulation of the rectal spacer in our previous study (Hamed 30, 39). These studies prove the great potential and versatility of FEMOSSA as a patient-specific spacer simulation algorithm. Not only can it be applied to other anatomical locations like head and neck spacers (24), but also it can be of great interest to both physicists and physicians to gain better insight into the mechanics of soft-tissue and hydrogel interaction in plastic surgery (40), drug (41), and biomaterial (42) delivery.

Taking advantage of FEMOSSA allowed us to do a systematic study of the correlation between spacer location and spacer benefits that is infeasible in practice. The result was used to develop a DSS to help health professionals make the most informed clinical decision and potentially spare the patients from unnecessary trauma of an invasive endoscopic ultrasound (EUS)-guided procedure and reduce the cost and time of treatment.

We validated FEMOSSA by quantifying the separation between HOP and duodenum in the complex C-loop-like shape of the duodenal loop using newly defined RNND and OVH metrics. The RNND profile can further be used as informative, quantified feedback to guide the EUS-based spacer injection procedure as it provides a 2D radial measurement of separation similar to the radial EUS viewpoint. The OVH is a scalar, on-demand metric that quantifies the 3D relative geometry of ROIs. Previously, it has been shown to have a high correlation with plan dosimetric indices (34, 43) and used to predict objectives and constraints, for automatic or semiautomatic treatment planning (44–47).

Our result showed that OVH *L*1*_CC_* has a very high correlation with duodenal high-dose volume (V33Gy). Given that our analysis suggests V33Gy was the main limiting factor to achieve target objectives and is highly sensitive to spacer location, we believe that L1cc is an informative preoperative and intraoperative anatomical feedback to guide the spacer procedure. Moreover, it indicates that OVH L1cc can be a good factor for automatic treatment plan optimization. These results justify the use of L1cc as quantified feedback, sensitive to adjacent OARs anatomy and spacer location, to create the most informed DSS.

The DSS was designed based on an NN-based predictive model and a Bayesian regression model. The NN method is a fast, relatively simple method to model multivariable non-linear relationships. The advantage of the Bayesian model is that instead of a point estimation of parameters, a probability distribution is estimated and, therefore, incorporates high variability of data by resampling the parameters. More importantly, both the NN and Bayesian methods have transfer-learning advantage that gives the model the ability to get updated with the newly presented data.

Our study has a few limitations. First is the limited number of cases for the validation phase. The duodenal hydrogel spacer is a very novel procedure and not yet widely used in the clinic. Previous studies done by our group have used a small volume of hydrogel spacer (<5 cc) which only results in a small separation (<2 mm) (13; Avani Dholakia 23). In recent clinical trials, larger hydrogel volume (<10 cc) was injected to achieve more separation and thus better PTV coverage, but the number of clinical trials is very limited. To undermine the effect of this limitation, we evaluated the performance of our model rigorously with three figures of merit, namely, DSC, OVH, and RNND. Moreover, by using an advanced physical-based model, we further ensured that the simulation is based on anatomical properties and is realistic.

Another limitation of the study is the uncertainties of the spacer placement process that can be a possible source of error. Uncertainties such as day-to-day variations of organ shape (like change in abdominal filling), organ contours, and tumor volume change due to concurrent chemotherapy make predicting the exact shape and effect of hydrogel spacer nearly impossible. We addressed this issue by using the Bayesian model for the prediction model that allowed the use of the Gibbs sampling method that in turn resulted in incorporating the uncertainty in our model and creating an empirical distribution of the data, and therefore a stochastic model. The Bayesian regression model generates an interval estimation of the parameters as opposed to point estimation, and therefore it allowed us to incorporate a higher level of uncertainty into the model’s prediction.

Finally, another limitation of our study is that, although FEMOSSA can create a patient-specific and realistic simulation of the hydrogel spacer, using the finite element method results in a long computation time. Nevertheless, with recent optimizations of our algorithm, we reduced the time from 2 h to less than 30 min on a desktop computer. Moreover, here we showed the feasibility of using FEMOSSA-generated augmentation to create real-time models like the designed DSS and artificial-intelligence-based models that require a large number of training data but can provide instantaneous output. Using a real-time model will further reduce the uncertainties as it can be used intraoperatively and thus minimize the effect of anatomy change.

## 5 Conclusion

In this study, we extended the application of FEMOSSA to the duodenal spacer, and using the simulated augmented data, we developed a DSS to provide preoperative patient selection and thus guidance for optimal location of the spacer. We found that spacer benefit for a high-dose volume is highly dependent on the patient’s anatomy and spacer location. Future work focuses on (1) improving the software and reliability of the model by incorporating a larger patient cohort, (2) adding more features to the DSS such as prediction of toxicity and cost-effectiveness, and (3) proposing a new workflow featuring preoperative simulation and intraoperative guidance to personalize and optimize the duodenal spacer procedure based on our studies on wavelet-based image guidance (25, 48).

## Data Availability Statement

The raw data supporting the conclusions of this article will be made available by the authors upon request.

## Ethics Statement

The studies involving human participants were reviewed and approved by the Johns Hopkins Medicine Institutional Review Boards. The patients/participants provided their written informed consent to participate in this study.

## Author Contributions

The study was designed by all authors. HH and KD prepared the manuscript. HH, SY, and KD contributed to data analysis and interpretation. HH, KD, AN, and ES participated in collecting data. All authors contributed to the article and approved the submitted version.

## Funding

Research reported in this publication was supported by the National Institutes of Health (award numbers R37CA229417). The content is solely the responsibility of the authors and does not necessarily represent the official views of the National Institutes of Health.

## Conflict of Interest

The authors declare that the research was conducted in the absence of any commercial or financial relationships that could be construed as a potential conflict of interest.

## Publisher’s Note

All claims expressed in this article are solely those of the authors and do not necessarily represent those of their affiliated organizations, or those of the publisher, the editors and the reviewers. Any product that may be evaluated in this article, or claim that may be made by its manufacturer, is not guaranteed or endorsed by the publisher.
