# Supplementary material for: Finite Element-Based Personalized Simulation of Duodenal Hydrogel Spacer: Spacer Location Dependent Duodenal Sparing and a Decision Support System for Spacer-Enabled Pancreatic Cancer Radiation Therapy
Source: Front Oncol. 2022 Mar 24;12:833231. doi: 10.3389/fonc.2022.833231 (PMC8987290; doi:10.3389/fonc.2022.833231)
Supplement: Supplementary file 1 [file Presentation_1.pptx]

## Slide 1
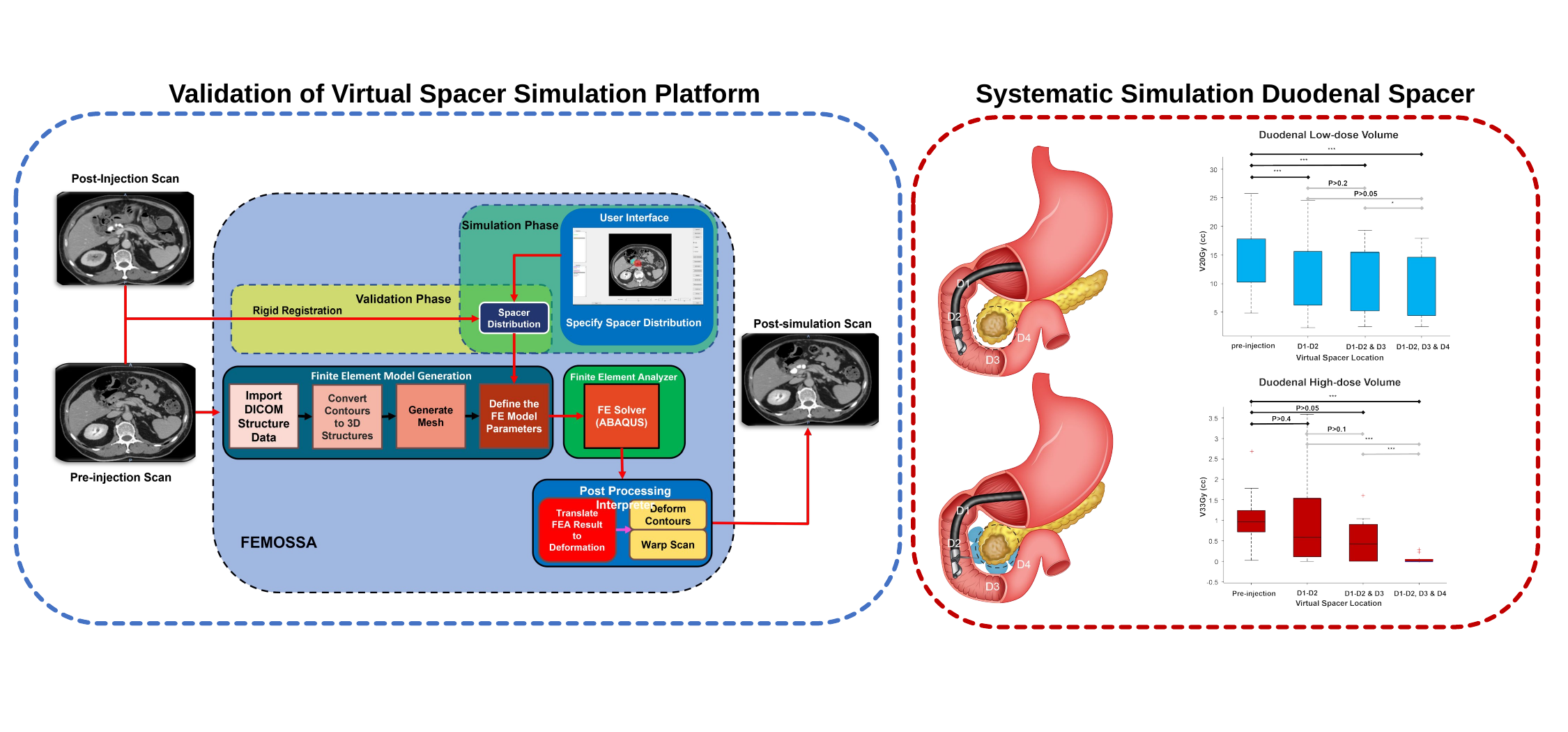

Validation of Virtual Spacer Simulation Platform
Systematic Simulation Duodenal Spacer
